# Safety evaluation of topical applications of ethanol on the skin and inside the oral cavity

**DOI:** 10.1186/1745-6673-3-26

**Published:** 2008-11-13

**Authors:** Dirk W Lachenmeier

**Affiliations:** 1 Chemisches und Veterinäruntersuchungsamt (CVUA) Karlsruhe, Weissenburger Strasse 3, D-76187 Karlsruhe, Germany

## Abstract

Ethanol is widely used in all kinds of products with direct exposure to the human skin (e.g. medicinal products like hand disinfectants in occupational settings, cosmetics like hairsprays or mouthwashes, pharmaceutical preparations, and many household products). Contradictory evidence about the safety of such topical applications of the alcohol can be found in the scientific literature, yet an up-to-date risk assessment of ethanol application on the skin and inside the oral cavity is currently lacking.

The first and foremost concerns of topical ethanol applications for public health are its carcinogenic effects, as there is unambiguous evidence for the carcinogenicity of ethanol orally consumed in the form of alcoholic beverages. So far there is a lack of evidence to associate topical ethanol use with an increased risk of skin cancer. Limited and conflicting epidemiological evidence is available on the link between the use of ethanol in the oral cavity in the form of mouthwashes or mouthrinses and oral cancer. Some studies pointed to an increased risk of oral cancer due to locally produced acetaldehyde, operating via a similar mechanism to that found after alcoholic beverage ingestion.

In addition, topically applied ethanol acts as a skin penetration enhancer and may facilitate the transdermal absorption of xenobiotics (e.g. carcinogenic contaminants in cosmetic formulations). Ethanol use is associated with skin irritation or contact dermatitis, especially in humans with an aldehyde dehydrogenase (ALDH) deficiency.

After regular application of ethanol on the skin (e.g. in the form of hand disinfectants) relatively low but measurable blood concentrations of ethanol and its metabolite acetaldehyde may occur, which are, however, below acute toxic levels. Only in children, especially through lacerated skin, can percutaneous toxicity occur.

As there might be industry bias in many studies about the safety of topical ethanol applications, as well as a general lack of scientific research on the long-term effects, there is a requirement for independent studies on this topic. The research focus should be set on the chronic toxic effects of ethanol and acetaldehyde at the point of impact, with special regard to children and individuals with genetic deficiencies in ethanol metabolism.

## Introduction

Ethanol is widely used as a solvent both in the home and in industry [1]. Consumers may be exposed to ethanol from its application as a constituent of many household and personal products, such as cosmetics, hairsprays, window cleaners, de-icers and certain pharmaceutical preparations [2]. Most people have experienced skin contact with alcoholic solutions [1].

The safety of topical applications of ethanol is still a matter of debate, and there appears to be scientific evidence pointing in both directions. On the one hand, researchers came to the conclusion that the range of damage caused to the skin by the alcohol cannot and should not be ignored, although the deleterious effects of ethanol exposure on the skin may pale into insignificance compared to its effects on the liver, central nervous system, and other body systems after ingestion [3]. On the other hand, scientific studies attributed ethanol for topical uses as safe *per se *[1,4-7]. However, there appears to be at least some evidence, including epidemiological data, about mouthwash use, and data from animal experiments showing that ethanol on the skin or inside the oral cavity may cause harm if used chronically. Evaluation according to EU cosmetics legislation [8] and other acts about chemical safety should consider the chronic toxic and carcinogenic potential of ethanol. In this article, the safety of topical uses of ethanol will be evaluated by a critical review of the scientific literature.

## Methods

Data on the safety of topical ethanol were obtained by a computer-assisted literature search using the key words "topical ethanol", "topical alcohol", mouthwash, mouthrinse, "hand disinfectant", "alcohol based disinfectant" "alcohol/ethanol & melanoma", "alcohol/ethanol & skin" "alcohol/ethanol & penetration", "alkanol permeation", "acetaldehyde & skin". Searches in both English and German were carried out in July 2008, in the following databases: PubMed, Toxnet and ChemIDplus (U.S. National Library of Medicine, Bethesda, MD), Web of Science (Thomson Scientific, Philadelphia, PA), IPCS/INCHEM (International Programme on Chemical Safety/Chemical Safety Information from Intergovernmental Organizations, WHO, Geneva, Switzerland), and Scopus (Elsevier B.V., Amsterdam, Netherlands). This was accompanied by a hand search of the reference lists of all articles for any relevant studies not included in the databases. The references, including abstracts, were imported into Reference Manager V.11 (Thomson ISI Research Soft, Carlsbad, CA) and the relevant articles were manually identified and purchased in full text.

## Review

Most research on ethanol is centred around its effects after ingestion in the form of alcoholic beverages, which is a major risk factor for the burden of disease in our society [9]. Significantly less information is available on the effects of ethanol if topically used on human skin or in the oral cavity. Our discussion will begin with the mechanisms of toxicity known from ethanol ingestion, for which there is evidence that they could also apply to topical ethanol use (i.e. carcinogenicity and local effects of ethanol on the human skin). After that, the effects of ethanol as a skin penetration enhancer will be discussed, which are excellently described from pharmaceutical applications. Finally, certain groups of products are discussed in detail (cosmetics, mouthwashes, and hand disinfectants), and an overall risk assessment is provided.

### Carcinogenicity of ethanol: is there a possibility of skin cancer after topical application?

The recent evaluation of ethanol in alcoholic beverages as 'carcinogenic to humans' must be considered in the risk assessment of topical application forms. This paragraph summarizes the scientific proof for this association, which has been primarily derived from epidemiological studies about the ingestion of alcoholic beverages.

In February 2007, the WHO's International Agency for Research on Cancer (IARC) re-assessed the carcinogenicity of alcoholic beverages in the context of the IARC monographs programme. 'Ethanol in alcoholic beverages' was classified as 'carcinogenic to humans' (Group 1) [10,11]. Overall, the IARC concluded that the occurrence of malignant tumors of the oral cavity, pharynx, larynx, esophagus, liver, colorectum, and female breast is causally related to alcohol consumption [11]. Because the associations were generally noted with different types of alcoholic beverages, and in view of the carcinogenicity of ethanol in animals, the IARC now considers ethanol itself (not other constituents or contaminants) as causative of the carcinogenicity of alcoholic beverages.

Many studies of different design and in different populations around the world have consistently shown that regular alcohol consumption is associated with an increased risk of cancers of the oral cavity, pharynx, larynx, and esophagus [12]. Daily consumption of around 50 g of alcohol (ethanol) increases the risk of these cancers by two to three times compared to non-drinkers [11,13-15].

Furthermore, in populations that are deficient in the activity of aldehyde dehydrogenase, an enzyme involved in the catabolism of ethanol, much higher risks for oesophageal cancer after alcohol consumption have been reported than in populations with a fully active enzyme [16]. This is also proof that acetaldehyde derived from ethanol metabolism contributes to its carcinogenicity. Results of animal experiments have confirmed the carcinogenicity of acetaldehyde and ethanol [11].

During topical-application of ethanol, the most prone organ for adverse effects appears to be the skin, which comes into direct contact with the agent. The second organ that may be regularly exposed to topical ethanol is the oral cavity through use of alcohol-containing mouthwashes or mouthrinses.

In their evaluation of the carcinogenicity of alcoholic beverages and ethanol, the IARC also appreciated the association between melanoma and alcohol consumption [10]. The IARC considered two cohort studies, one in an occupational group exposed to ionizing radiation and one in alcoholic women. In the cohort study of radiologic technologists in the U.S.A. and in the study of alcoholic women in Sweden, no significant associations were seen [17,18]. Furthermore, a number of case-control studies published results on melanoma risk in relation to alcohol intake. Some of those studies reported no significant association between alcohol intake and melanoma risk [19-23]. Whereas, three case-control studies in the U.S.A. reported some increase in risk of melanoma associated with alcohol intake [24-26]. None of these were adjusted for exposure to UV light, and thus the possibility of confounding cannot be excluded. The IARC concluded that melanoma is not one of the cancer sites with a clear association with ethanol consumption. Besides melanoma, a few studies have linked alcohol consumption to a higher risk of basal cell carcinoma [27,28].

Only a few studies have suggested potential biological mechanisms for a possible relationship between alcohol and melanoma risk [17]. The high-risk behaviour of binge and heavy drinking may be associated with higher rates of sunburn, which may lead to skin cancer [29]. A pituitary-mediated mechanism has been proposed as a direct effect of ethanol [30,31]. Another hypothesis on the aetiology of alcohol induced melanoma is an altered redox state caused by alcohol metabolism [32]. Ethanol ingestion may also lead to a decrease of carotenoid antioxidant substances in the skin, which then causes erythema to occur faster and with greater intensity following UV irradiation [33].

Interesting evidence into the induction of melanoma and non-melanoma skin cancers is provided by the animal experiments of Strickland et al. [34-36]. The studies suggest that the interaction of topically applied compounds like ethanol and Aloe emodin (a trihydroxyanthraquinone found in *Aloe barbadensis*), may be, in conjunction with UV radiation, important in causing melanin-containing tumours. As an underlying mechanism the authors speculated that the anaerobic flora of the pilosebaceous unit transforms ethanol to acetaldehyde and thus fosters ethanol-based carcinogenesis. The authors found that their research may pose public health implications due to the presence of these compounds in consumer products, especially the simultaneous use of ethanol and the gel of *Aloe barbadensis*, which forms the basis of a large number of skin care products, under exposure of UV light. However, it remained undetermined if the results from animal experiments may be transferable to humans.

All in all, it can be concluded that there is a lack of evidence to associate topical ethanol use with an increased risk of skin cancer. However, the carcinogenic properties of ethanol must be regarded in the risk assessment of such products anyway, because ethanol may be transported by the blood stream to more susceptible organs after skin penetration (see below). The synergistic effects with *Aloe barbadensis *show that each formulation of an ethanol containing product must be thoroughly evaluated for its carcinogenic potential.

### Other effects of ethanol on the skin

Besides skin cancer, alcohol abuse has been associated with the development of several skin disorders including psoriasis, discoid eczema and superficial infections [37-40]. Chronic alcohol abuse is also a predisposing factor for necrotizing wound infections, delayed wound healing and cellulitis [41]. There are several theories about the causes for such skin diseases including immune suppression, mal-nutrition, liver disease [42] or the influence of alcohol on lipid metabolism [43]. As acute and chronic alcohol abuse modulate immunity [44], this mechanism can explain dermatological diseases, which have an immune pathogenetic mechanism [42]. However, there are only a few studies about the molecular mechanisms of alcoholic skin diseases. Farkas et al. [45] determined a stimulatory effect of ethanol on human keratinocytes, which may be one of the reasons why psoriasis can be precipitated by alcohol misuse.

Topical application of 10% ethanol stimulates the proliferation of peritoneal tissue explants – a semi in-vivo wound model – which can be interpreted as positive influence for stimulation of wound healing by ethanol [46].

An interesting patch test was conducted by Haddock et al. [47]. 1.5-cm patches moistened with 0.1 ml of 100% ethanol or 10% acetaldehyde were applied to a group of patients. No erythema were observed from patch tests with ethanol on non-hydrated skin, while all applications of acetaldehyde resulted in notable erythema. Using the same test on hydrated skin (i.e. immersion of the test site in water for 10 min before application of the patches), localized erythema were also caused by ethanol. The reactions were judged to represent a direct pharmacologic action of topical alcohols on the cutaneous microvasculature, and that erythemogenesis is enhanced after hydration because of an increase in cutaneous permeability to alcohol.

Höök-Nikanne et al. [48] found that very high acetaldehyde levels up to 960 μmol/l were formed *in vitro *by different bacteria strains typically found on the human skin at ethanol concentrations known to exist in sweat during normal social drinking. The authors concluded that this primary observation of bacterial production of acetaldehyde could offer an explanation for the deleterious effect of alcohol on various skin diseases, and that these preliminary results warranted further *in vivo *study. However, to our knowledge no further studies into this mechanism were conducted. This research would be extremely important, as the formation of acetaldehyde either by bacteria strains on the human skin or by metabolism following absorption is also a likely mechanism in topically applied products. However, the amount of acetaldehyde formation after topical application of ethanol on intact, healthy skin is currently unknown. The bacterial acetaldehyde production may be restricted as both the transient and resident microorganisms may be significantly reduced by the ethanol application, which should lead to higher local ethanol concentrations as in the case of systemic distribution after alcohol ingestion. In addition, the contact time should be shorter in the case of topical ethanol application because of the fast evaporation of the alcohol.

### Ethanol as a penetration enhancer

Systematic *in vitro *and *in vivo *studies have elucidated the mechanism of percutaneous alcohol absorption [1,49-62]. Numerous data are available on permeability, partition coefficients and diffusion constants. It is now generally accepted that the "barrier" function of the skin resides almost entirely in the stratum corneum [53,55,63,64]. Most water-soluble, low-molecular weight non-electrolytes – among them ethanol – applied to the skin surface can diffuse much faster into the blood-stream if the epidermis is diseased, damaged or removed [63].

Ethanol is also well known as a topical penetration enhancer and may be used in transdermal delivery systems [65-81]. Bommannan et al. [82] found *in vivo *in humans that ethanol enters the skin and removes measurable quantities of the lipid barrier material from the stratum corneum. This lipid extraction may lower the skin barrier function and render the membrane more permeable, which is the most likely explanation for the effect of ethanol as a skin penetration enhancer. Kai et al. [83] and van der Merwe et al. [84] confirmed those results. Goates et al. [85] additionally remarked that enhanced permeation may be caused not only by extraction of lipids but also of proteins from human skin in the presence of aqueous alcohol solutions. The mechanism of ethanol as a skin permeation enhancer was described to be a so-called 'pull' or 'drag' effect, which means that the permeation of the enhancer subsequently facilitates that of the solute (in the sense of a simple co-permeation) [79,80]. Side-effects of the transdermal patches were cutaneous reactions, where ethanol proved to be one of the causes of cutaneous intolerance or allergic contact dermatitis [86-89]. However, in some of these cases combination effects between the different constituents of the preparation cannot be excluded, so that it remains unclear if ethanol or other impurities were the real cause for the allergic effects observed.

Animal studies demonstrated that both chronic and acute ethanol consumption increase transdermal penetration, resulting in higher exposure of several xenobiotics, e.g. herbicides [90-92] or the tobacco carcinogen nitrosonornicotine [93]. The transdermal absorption of xenobiotics may be facilitated by ethanol induced changes in lipid peroxidation and transepidermal water loss (TEWL) [41,94]. In contrast, the influence of orally administered ethanol on TEWL did not affect the penetration of a topically applied UV filter substance [95]. Changes in TEWL were not only detected after ingestion of ethanol, but also after topical application [77,96]. In contrast, other studies found that there is no transepidermal water loss after topical ethanol application [97,98].

### Blood alcohol levels after ethanol absorption through skin

The previously mentioned studies about ethanol as a penetration enhancer for pharmaceutical preparations show that ethanol is absorbed into the normal, intact skin, and may reach the blood stream to be systemically distributed in the human body.

Anderson et al. [99] also confirmed these results using microdialysis techniques, which showed that percutaneous absorption of alcohols can occur through intact skin.

Bowers et al. [100] reported a controlled study to assess the likelihood of ethanol being absorbed through intact skin and producing measurable blood-ethanol concentrations in experiments involving four children (7–9 years of age) and one adult. The legs of the subjects were wrapped in cotton from above the knees to the feet, and the wrappings were subsequently soaked with 200 ml of 95% (v/v) ethanol. Although the ethanol-soaked cotton was kept covering the skin with rubber sheeting and adhesive tapes for 4–9 hours, no ethanol was measurable in the blood.

Schaefer and Redelmeier [6] estimated the percutaneously absorbed dose of ethanol from a topical application. Using Scheuplein and Blank's [54] permeability coefficient, a skin exposure area of 1000 cm^2^, and assuming a maximum exposure period after topical application of significantly less than 1 hr, they estimated that the percutaneous absorption of ethanol from a 70% solution would be approximately 100 mg. Schaefer and Redelmeier equated this amount of ethanol to that present in 1.5 ml of wine containing 10% (v/v) ethanol, and therefore concluded that "skin exposure to ethanol in cosmetics is not a safety concern".

To our knowledge, the only study in the literature about blood alcohol concentrations in humans after use of cosmetics on the skin (alcohol based deodorant spray) was conducted by Pendlington et al. [1]. Sixteen adults sprayed an aerosol containing 44% ethanol over the body for approximately 10 sec (mean amount used per treatment: 9.72 g). Blood samples were taken after a 15 min period. Subsequent samples were taken 5, 10, 30 and 60 min after that. Ten of the panellists produced at least one blood sample with a detectable alcohol content (detection limit: 5 mg/l). The maximum value recorded was 13 mg/l. However, there remained some uncertainty in the analytical method, as other alcohols may co-elute. Using another gas chromatographic column (detection limit: 9 mg/l), none of the blood samples exhibited detectable levels of ethanol. The application as a spray also includes a potential pulmonary uptake. Despite the high concentration of ethanol (44%) and the high exposure to large surfaces, the maximum blood levels were only slightly elevated above physiological blood levels (average 0.4 mg/l [101]).

More information is available about the blood alcohol concentrations arising from the use of alcohol-based disinfectants. Miller et al. [102] reported the blood alcohol level after using an alcohol-based instant hand sanitizer (62% (v/v) ethanol) under most extreme conditions (applying 5 ml, 25 times over the course of 2 hours). The blood alcohol level measured immediately following the final application was below the detection limit (< 5 mg/dl). In a subsequent study of 5 subjects using 5 ml of the product with a repetition of 50 times over 4 hours, the result was confirmed as all participants had blood ethanol levels less than 5 mg/dl. No adverse reactions were noted during the study [103]. The major constraint of the studies of Miller et al. [102,103] is the relatively high detection limit. Subsequent studies with more sensitive methods showed that in fact detectable blood ethanol concentrations may arise after using hand disinfectants. However, the concentrations were judged by the authors as being below acute toxic levels, i.e. ethanol was unable to cause adverse effects within a short time of dosing or exposure (acute and chronic toxicity are used according to IUPAC definitions throughout the text [104]).

In the study of Kirschner et al. [5] with a detection limit of 0.5 mg/l, serum ethanol concentrations in the range of 1.0–1.5 mg/l were detected after application of 20 ml of alcohol-containing disinfectant (74.1% ethanol) on a 200-cm^2 ^gauze swab for 10 min. The exclusion of inhalative uptake was given as rationale for the lower concentrations in comparison to other studies. The dermal uptake of ethanol was judged by the authors to be clinically insignificant. In the study of Kramer et al. [4], 12 volunteers applied three hand-rubs containing 95% (w/w), 85% (w/w) or 55% (w/w) ethanol. 4 ml were applied 20 times for 30 s, with a 1 minute break between applications. The highest median concentrations found were 20.95 mg/l, 11.45 mg/l and 6.9 mg/l, respectively. The proportion of absorbed ethanol was 1365 mg (2.3%), 630 mg (1.1%), and 358 mg (0.9%), respectively. In addition, blood acetaldehyde was determined, the highest median of which was 0.57 mg/l. It can be concurred with the authors that acute toxic effects cannot be expected even after excessive use of ethanol-based disinfectants. An impairment of performance is usually assumed from blood ethanol concentrations of 200–300 mg/l and above [105]. Therefore, the concentrations achieved by hand disinfectant use are at least a factor of 10–20 below the values required for acute toxicity. However, it is difficult to agree with Schaefer and Redelmeier [6], Kirschner et al. [5] and Kramer et al. [4] that the use of cosmetics or ethanol-based hand rubs is "safe" *per se*. The chronic toxic effects of ethanol and acetaldehyde have certainly to be accounted for in the safety evaluation of topical ethanol applications. This was done in neither of the above mentioned studies about the toxicity of skin disinfectants.

### Ethanol absorption through lacerated skin: a health risk especially for children

The possibility of alcohol absorption across the injured skin is generally accepted in the literature [63]. In 1950, Paulus [106] conclusively showed in animal experiments that alcohol is absorbed relatively rapidly through areas of wounded skin. A human case relating to the absorption of ethanol through abraised and lacerated skin was reported by Jones et al. [107]. The damaged skin (33% of total body surface) of a victim of a traffic accident was washed in the operating theatre with surgical spirit (70% (v/v) ethanol). A blood ethanol concentration of 0.046 g/100 ml was determined, which corresponded to an absorption of approx. 30 ml of the ethanol solution. The authors concluded that there is a risk of ethanol being absorbed into the bloodstream if damaged skin is washed with surgical spirits, which may have ramifications in civil litigation (e.g. responsibility for accidents, insurance claims).

Alcohol is an agent that poses a risk of percutaneous toxicity in the newborn. Exposure of immature skin (especially under occlusion) may lead to significant local reactions and systemic toxicity [108]. Percutaneous absorption of ethanol through damaged skin resulting in clinical manifestations of intoxication has been reported in a 1-month-old infant [109] and in a 2-year-old child [63]. Giménez et al. [110] reported ethanol poisoning in 28 children, aged one to 33 months, after application of alcohol-soaked cloths to relieve abdominal pain (which was a common practice in Argentina). Two of the children with ethanol poisoning died. A fatal intoxication due to percutaneous ethanol absorption in an infant was also described by Niggemeyer et al. [111]. Skin necrosis and elevated blood alcohol levels have also been observed in preterm infants [112,113], whose immature, poorly keratinized skin is an ineffective barrier to potentially toxic compounds such as alcohol. In the case of the child intoxication mentioned above, the damage to the epidermis accounted for an alcohol absorption rate approximately 1000 times faster than that across intact stratum corneum [63].

Based on all scientific evidence alcohols including ethanol are not recommended for use on abraised and lacerated skin, and due to the expected burning sensation also not for a cosmetic application.

### Ethanol in mouthwashes and oral rinses

Ethanol is still a component of a significant number of oral-care products [114]. When adults use such ethanol-containing mouthwashes, oral rinses, and similar products as they are intended to be used, an acute-toxic effect in the sense of typically intoxication occurring after alcoholic beverage consumption caused by an increased blood-alcohol level is not likely (note: the abusive ingestion of products intended for topical use will not be considered in this article; please refer to references [115-119]).

The absence of acute-toxic effects in adults has previously been interpreted to indicate that such mouth-rinsing cosmetics are safe in every respect. However, the risk arising from this product group does not result primarily from systemic blood alcohol concentrations, but emanates from the locally formed acetaldehyde (see section 'Carcinogenicity of ethanol' above). Further adverse effects of the use of mouthwash were reviewed by Gagari et al. [120]. For adults, these are predominantly local and systemic allergic effects, which were postulated to be caused by the combination of a high content of alcohol, an acidic pH, and other ingredients that act individually or synergistically. Furthermore, it was shown that the *in vitro *toxicity of ethanol-containing mouthwashes may exceed that of pure-ethanol solutions [121]. Whereas, other *in vitro *tests failed to detect mutagenic or carcinogenic hazards of mouthwashes [122]. Other studies also reported the opposite effect that ethanol containing mouthwashes may be less toxic than formulations without ethanol in tissue cultues of explants of neonatal rat peritoneum [123].

However, another recent study showed that the genotoxicity of mouthwashes is caused by ethanol and not by any other ingredient [124]. This is in line with mechanistic evidence summarized by the IARC that ethanol causes sister chromatid exchange in both lower organisms and mammalian cells, including human cells, and that the data from studies in animals suggest that ethanol causes DNA damage in target tissues [10].

**Figure 1:**
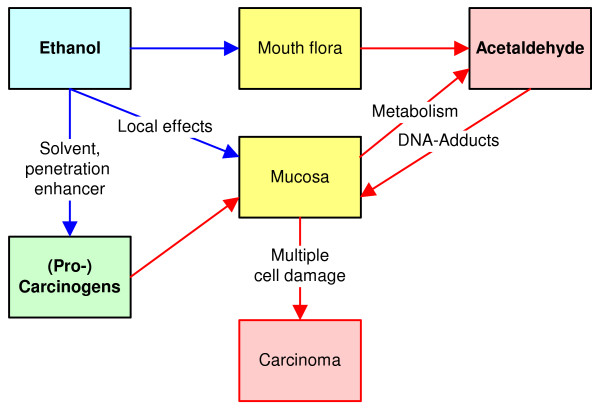
Simplified model of the mechanism of carcinogenesis in the oral mucosa after using ethanol-containing mouthrinses.

Mechanistic evidence especially points to detrimental effects of ethanol in the upper gastrointestinal tract (i.e. the oral cavity, pharynx, larynx/hypopharynx). The mucosa may be damaged by ethanol, which leads to the stimulation of cell regeneration. Genetic changes may then cause the development of dysplasia or leukoplakia and, finally, cancer [125,126]. The possibility of damage to the oral mucosa also exists with the use of mouthwashes [127]. An overview of the effect of ethanol on the oral mucosa is shown in Figure 1. Local damage to the mucous membrane also facilitates the development of tumours on such exposed locations by the increased absorption of other carcinogenic substances. Besides acetaldehyde, the microsomal metabolism of ethanol leads to reactive oxygen species, which can also covalently bind to the DNA [128]. Although the liver represents the major site for cytochrome P450 (CYP) dependent metabolism, extrahepatic tissues including the buccal mucosa may express CYP activity [129,130]. The contributions of the different metabolic pathways to ethanol oxidation in the oral mucosa after mouthwash consumption are currently unknown. Besides the metabolic conversion of ethanol in human cells, we have to consider oxidation of ethanol into toxic acetaldehyde by microorganisms in the oral cavity and the pharynx, which can be found in a physiologically massive density [131-133]. It is remarkable that many of the oral rinses found on the market have a higher alcoholic strength than, for example, beer. Therefore, the possibility of a very high acetaldehyde concentration in the saliva arises, even without ingestion of the product (see below). For further information on the molecular mechanisms of the carcinogenicity of alcohol, the current review article of Seitz et al. [134] is recommended.

Epidemiological studies on the link between mouthwash use and oral cancer risk were recently reviewed by La Vecchia [135]. From the 10 case-control studies published over the last three decades, three reported relative risks above unity and seven no consistent association. However, in many cases the study designs were flawed as they did not differentiate between alcohol-containing and alcohol-free mouthwashes. One example is the multicenter case-control study of Guha et al. [136] that indicated daily mouthwash use as cause for cancers of the head, neck and oesophagus, however, the association remains dubious because the alcohol content and duration of use were not recorded.

Two of the studies that differentiated between mouthwash types found that the risk was correlated to the alcoholic strength of the mouthwashes [137,138]. The risk was confined to users of mouthwash high in alcohol content (>25% vol) [137]. An non-significantly elevated risk was also observed among the small number of subjects who neither smoked cigarettes nor drank alcohol in a study conducted in Puerto-Rico [139]. Earlier studies also reported limited evidence that the use of mouthwash may be associated with an increase in the risk of oral cancer in groups such as non-smoking, non-drinking women who are ordinarily at a low risk [140,141].

**Figure 2:**
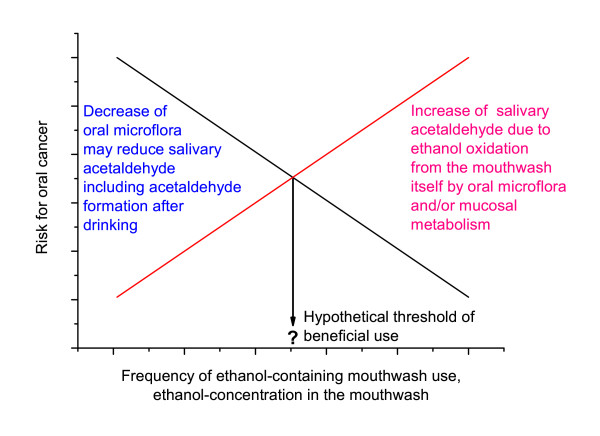
Hypothetical model for mouthwash related carcinogenic risk.

From these limited results, it may be hypothesized that the use of mouthwashes could have a threshold for adverse effects (Figure 2). It is known that oral hygiene may have an influence on risk for oral cancer [142], so the use of mouthwash could reduce the acetaldehyde-producing oral microflora. However, there still exists the possibility for metabolic acetaldehyde production directly in the mucosa by alcohol dehydrogenase.

According to Eriksson, the salivary acetaldehyde represents mostly microbial acetaldehyde formation in the oral cavity, but also, to some extent, ethanol oxidation in nearby tissues [143]. *In vivo *acetaldehyde production after ethanol consumption is significantly reduced after a 3-day use of an antiseptic mouthwash (chlorhexidine) [144,145]. There are currently many research gaps regarding mouthwash use. The analysis of the microbial flora appears to be necessary for interpretation of acetaldehyde levels in saliva after mouthwash use as well as the long term measurement of acetaldehyde levels, if alcoholic and non-alcoholic antimicrobial mouthwashes are used.

Further research into the molecular mechanism of mouthwash mediated oral cancer is also needed. Furthermore, the epidemiological evidence appears inadequate so far [135,146-150], and larger case-control studies are necessary that clearly differentiate between the different types of mouthwash.

However, on this stage the currently available data provide, at the least, doubts about the general safety of alcohol-containing oral products. It appears to be prudent precautionary public health policy to generally refrain from using ethanol in such products. For example, the Centers for Disease Control and Prevention (CDC) stated that although there is no certain link between oral cancer and mouthwash, its excessive use should be discouraged [151].

It has been demonstrated a number of times that alcohol-free oral rinses are as effective as their alcohol-containing counterparts, and therefore the necessity for ethanol in mouthwashes and oral rinses appears to be non-existent [152-154]. Products without alcohol have also been shown to have a lower incidence of other adverse effects [155].

### Ethanol in hand disinfectants

Ethanol-based hand disinfectants are widely used in occupational settings not only in hospitals but also in all other areas that demand hand-hygiene (e.g. food production). The antimicrobial effects of alcohols (except methanol) are based on protein denaturation [46]. Alcohols have excellent, and the most rapid bactericidal and fungicidal activity of all agents used in hand disinfection [156]. In terms of antimicrobial efficacy, 1-propanol can be regarded as the most effective alcohol, followed by 2-propanol and ethanol [156]. Comparison of 2-propanol with ethanol showed that the efficacy of 2-propanol 60% (v/v) is almost equivalent to ethanol at 80% (v/v) [157]. Nevertheless, ethanol was described to be preferred because the smell of isopropanol (2-propanol) was considered unacceptably disagreeable [158]. However, the smell of a substance is of course toxicologically irrelevant and should therefore not be a criterion to choose ethanol. While alcohol-based hand rubs generally have a broad and relatively rapid activity against vegetative bacteria, they are often limited in their ability to inactivate non-enveloped viruses [159].

There is no unanimous view on the safety of ethanol-based hand disinfectants in the scientific literature:

• On the one hand, alcohols were described as non-toxic in their application as a hand disinfectant and they were judged to lack any allergenic potential [156]. It was also concluded that alcohol-based hand rubs have a less deleterious effect on the skin than other physical irritants, which enhance skin reactivity [160]. The repetitive use of different alcohol-based hand rubs was shown to not significantly change transepidermal water loss, dermal water content or the sebum content of the skin [98]. The potential of ethanol-containing hand rubs to cause skin irritation was tested using single and repetitive patch tests and wash tests. No significant change in skin barrier or erythema was induced, whereas skin hydration decreased significantly. The wash tests demonstrated that alcohol application caused significantly less skin irritation than washing with a detergent. Even on previously irritated skin, ethanol did not enhance irritation. Alcohol-based hand rubs cause less skin irritation than hand washing, and are therefore preferred for hand hygiene from the dermatological point of view [97].

• On the other hand, the previously mentioned experimental design used for evaluating the effects of alcohol-based hand rubs on the skin (i.e. patch testing with single alcohols) was criticized, because exposure to a wide variety of chemical irritants such as surfactants and detergents is frequent. The effects of simultaneous application of different irritants had been shown to induce significantly stronger reactions than those caused by application of each irritant on its own [160]. Irritation with alcohols is said to be common, and many healthcare workers complain about non-acceptable skin irritation caused by alcohol-based hand rubs [160]. Allergic contact dermatitis or contact urticaria syndrome induced by exposure to ethanol was previously described [86,87,161-175]. However, especially with the use of ethanol in hand disinfectants, the cause is not clear [159]. When reactions do occur, they may be caused by hypersensitivity to the alcohol itself, to aldehyde metabolites, or to some other additive of the topically-applied products [86].

The most likely cause for reactions to ethanol applied to the skin is the oxidative metabolism. Cytochrome P450, alcohol dehydrogenase, and aldehyde dehydrogenase (ALDH) activities have been demonstrated in skin [174]. However, large differences in genotype distribution were observed between different ethnic groups, with the non-functional ALDH2*2 allele being seen more commonly in Asian populations [176]. ALDH deficiency has been suggested to contribute to anaphylactic reactions to ethanol [173,174,177].

### Industry participation in studies about the safety of topically applied ethanol

Warnings can be found in the recent literature about systematic bias in scientific studies favouring products that are made by the company funding the research [178-180]. It became evident that a number of studies dealing with the safety of topically applied ethanol reviewed in this article (especially those about mouthwashes and hand disinfectants) were supported by industry, or at least one of the researchers was a paid employee of a manufacturer of the discussed product. The relevant studies are summarized in Table 1 according to the outcome and industry participation. It can be generally seen that the studies with industry participation judged ethanol to be safe *per se*, whereas independent studies were more cautious.

**Table 1:** Summary of articles about safety assessment of hand disinfectants and mouthwashes

Outcome of the study	Studies with no obvious industry sponsorship or participation	Studies with co-authors from industry or studies with declared industry financing
Positive outcome ("ethanol is safe", "no link between mouthwash use and oral cancer", "unlikely that mouthwashes increase risk of developing oropharyngeal cancer")	[150]	[1,4,5,135,146-148]

Negative or cautious outcome ("relationship between mouthwash use and oropharyngeal cancer", "conflicting findings in the literature", "mouthwashes probably irritate the oral mucosa", "further research needed")	[127,137-139,149]	

Patel [181] had previously questioned whether studies on hand disinfectants were flawed due to a conflict of interest, as one of the researchers was a paid employee of an alcohol hand rub manufacturer included in the trial, and the work was supported by grants from the manufacturer.

In the mouthwash studies, potential conflicts of interest were detected by Mascarenhas [149] in the re-analysis of Cole et al. [146] of the data from the National Cancer Institute provided in the study of Winn et al. [137]. The study of Cole et al. was financially supported by Warner-Lambert Company (the former maker of Listerine). It is interesting that from the same dataset, Winn et al. [137] concluded that there is a significantly increased risk of oral cancer associated with the regular use of mouthwash, but Cole et al. [146] concluded that this association is unlikely. The meta-analysis of Elmore et al. [147] financed by Procter & Gamble Co. equally detected no support for a link between mouthwash use and oral cancer. The recent review of La Vecchia [135] on mouthwash was conducted with partial unconditioned support from Johnson and Johnson Consumer (the current maker of Listerine).

As it was evident in other areas of research [180], industry supported reviews on ethanol should be read with caution, as they had more favourable conclusions than the corresponding independent studies. To analyze the research design of the industry-supported studies in question in more detail would have gone beyond the scope of the current article, so it remains uncertain if "industry bias" or other factors such as superior design can explain the differences in outcome of the studies. The possibility for bias, however, suggests the requirement of further independent research on alcohol-based hand disinfectants as well as mouthwashes.

### Legal aspects about ethanol and acetaldehyde in consumer products

Despite the above mentioned IARC evaluations, ethanol itself is not yet classified as carcinogenic in the context of European laws relating to dangerous substances [182]. Ethanol was also so far not evaluated by the Scientific Committee on Consumer Products. For this reason, the first metabolite of ethanol has to be used as a proxy because such information is available only for acetaldehyde.

According to the EU regulations on dangerous substances, acetaldehyde is categorized as a mutagenic and carcinogenic substance in category 3 (CMR 3) [182]. This is in accordance with the IARC that found sufficient evidence in animals to demonstrate carcinogenicity of acetaldehyde, and therefore evaluated the substance as possibly carcinogenic to humans also (group 2B) [183]. For those reasons, the EU's scientific committee on cosmetic products and non-food products intended for consumers (SCCNFP) has critically evaluated this substance [184]. Acetaldehyde is a constituent of many fragrance and flavour compounds and therefore is a minor component in a large number of cosmetic products (in the range between 0.1 and 2 mg/kg). The human exposure to acetaldehyde in cosmetic products was estimated by the SCCNFP to be 0.1 μg/kg bodyweight/day. Nasal carcinomas were detected during rat inhalation studies with acetaldehyde, and the threshold dosage was found to be HT25 = 36.7 mg/kg bodyweight/day, with which a neglectable lifetime cancer risk of 7E-7 may be calculated according to the T25-method of Sanner et al. [185]. The SCCNFP briefly acknowledges the carcinogenic effect of acetaldehyde as a metabolite of ethanol in the context of alcoholic beverages, but does not at all consider alcohol-containing products in its opinion on acetaldehyde. For this reason, the author thinks that it is likely that the SCCNFP has considerably underestimated the human exposure to acetaldehyde. The SCCNFP evaluation could also be criticized because it uses toxicological data from inhalation studies to assess dermal exposure.

The risk assessment of the SCCNFP was not implemented into the EU cosmetics directive 76/768/EEC [186]. However, the classification as a 'CMR 3 substance' explicitly demands the introduction of acetaldehyde into Annex III of the directive, because otherwise the substance would be prohibited according to Article 4b, as it had to be listed in Annex II of the directive. The risk management bodies of the EU are currently discussing a maximum authorized concentration of 20 mg/kg in the finished cosmetic product. Such a rule, however, would not be applicable to mouthwashes or most other consumer products because acetaldehyde is not contained in the products themselves, but only formed from ethanol during use in the oral cavity or on the skin. For this reason the maximum value in the European cosmetics directive cannot be used as a foundation for legal restrictions on alcohol-containing consumer products. Such restrictions would rather result from the safety evaluation of the products (see conclusions). Preliminary studies of mouthwashes have, for example, shown that acetaldehyde may be contained in concentrations up to 80 μmol/l in the saliva after rinsing with alcohol-containing mouthwashes, which was significantly above endogenous levels [187]. The salivary concentration may therefore reach the range of 40 to 200 μmol/l, which is already able to cause mutagenic or carcinogenic effects according to literature data [144,188].

All in all, there appears to be a legal void about the regulation of ethanol in consumer and medicinal products. Necessary future steps include the acknowledgment of ethanol's carcinogenic properties in the laws on dangerous substances, as well as the safety assessment in the framework of the laws about consumer and cosmetic products.

## Conclusion

The major conclusions of our literature review on the safety of topically applied ethanol are summarized in the Appendix.

The facts that ethanol is widely used in topical applications and that its adverse effects were seldom reported should not be dismissed. But a confounding factor in any study is the widespread use of alcoholic beverages. However, the prevalent consumption of alcoholic beverages in our society cannot be used as an excuse to completely negate any adverse effect of ethanol in cosmetic or other topical preparations, especially in occupational settings with high exposure to the ethanol containing products. As was pointed out in some of the studies reviewed in this article, the possibility exists that on the point of impact, very high concentrations of ethanol and acetaldehyde may cause chronic toxic effects. The effects may be more pronounced in ALDH-deficient humans, but this association demands further research.

Due to the conflicting evidence in many cases, the precautionary toxicological principle should be currently favoured in the evaluation of ethanol for topical uses. Until unambiguous evidence about the safety of ethanol in topical preparations exists, the necessity of its use should be critically evaluated. In certain product groups (e.g. mouthwashes), ethanol can be easily substituted for other compounds. In other product groups - especially hand disinfectants in hospital hygiene -, the advantages for the patients may outweigh the potential risks for the users. However, in this case, the formulations should be critically evaluated if ethanol cannot be at least partially substituted with e.g. other alcohols with a more favourable toxicological profile.

Assessment of cosmetic safety was introduced into European cosmetics law by Council Directive 93/35/EEC (amending for the sixth time Directive 76/768/EEC on the approximation of the laws relating to cosmetic products) [8]. This Directive is an important instrument in the protection of consumer health in terms of the use of cosmetic products. A re-examination and actualization of the safety assessment is necessary if scientific evidence concerning the ingredient employed in cosmetics changes [189]. With respect to the past years' scientific findings about the carcinogenic properties of ethanol, and the recent re-evaluation of this agent by the International Agency for Research on Cancer (IARC), it seems necessary to re-evaluate and actualize the safety assessment of topical products that contain this alcohol.

Finally, an advancement in testing strategies for genotoxicity and mutagenicity appears to be necessary [190], with a refocus on testing the final formulation rather than the isolated constituents [191]. The effect of ethanol as penetration enhancer for other constituents of the formulations must especially be considered in such a safety evaluation of cosmetics.

## Competing interests

The author declares that he has no competing interests.

## Authors' contributions

DWL conceived the study, conducted literature research and review, and wrote the first and final draft of the manuscript.

## Authors' information

DWL is state-certified food chemist and holds a doctorate in forensic toxicology. He currently heads the alcohol laboratory at the CVUA Karlsruhe, which is a governmental institute participating in surveillance of animals as well as food, cosmetic and pharmaceutical products aiming for public health and consumer protection. DWL has recently worked as expert in the IARC monographs working group Vol. 96 'Consumption of Alcoholic Beverages and Ethyl Carbamate (Urethane)'.

## Appendix

### Summary points and conclusions on the safety of topically applied ethanol

1. Topically applied ethanol (e.g. in the form of cosmetics or hand disinfectants) on un-lacerated human skin will not cause acute or systemic toxic effects, which can only occur if applied on damaged skin especially in children.

2. Adverse effects of topically applied ethanol may include skin irritations or allergic contact dermatitis.

3. Ethanol and its metabolite, acetaldehyde, are potentially carcinogenic for humans, however, only limited evidence supports the carcinogenicity of mouthwashes, and a complete lack of data about the carcinogenicity of all other groups of products (e.g. cosmetics, hand disinfectants) was detected.

4. Further concerns include the permeation-enhancing capabilities of ethanol, which could lead to an increased absorption of other components of topically applied formulations (e.g. nitrosamines from cosmetics).

5. Safety assessments of ethanol in any form of application must include the carcinogenic and genotoxic properties of ethanol and its metabolite acetaldehyde.
